# Complete Draft Genome Sequence of the Actinobacterium *Nocardiopsis sinuspersici* UTMC102 (DSM 45277^T^), Which Produces Serine Protease

**DOI:** 10.1128/genomeA.00362-17

**Published:** 2017-05-18

**Authors:** Bogdan Tokovenko, Christian Rückert, Jörn Kalinowski, Fatemeh Mohammadipanah, Joachim Wink, Birgit Rosenkränzer, Maksym Myronovskyi, Andriy Luzhetskyy

**Affiliations:** a Helmholtz Institute for Pharmaceutical Research Saarland, Saarbrücken, Germany; b Technology Platform Genomics, Center for Biotechnology (CeBiTec), Bielefeld University, Bielefeld, Germany; c Department of Microbial Biotechnology, School of Biology and Center of Excellence in Phylogeny of Living Organisms, College of Science, University of Tehran, Tehran, Iran; d Microbial Strain Collection, Helmholtz Centre for Infection Research, Braunschweig, Germany; e University of Saarland, Department of Pharmaceutical Biotechnology, Saarbrücken, Germany

## Abstract

The genome sequence of alkalohalophilic actinobacterium *Nocardiopsis sinuspersici* UTMC102 is provided. *N. sinuspersici* UTMC102 produces a highly active serine alkaline protease, and contains at least 11 gene clusters encoding the biosynthesis of secondary metabolites. The *N. sinuspersici* UTMC102 genome was assembled into a single chromosomal scaffold.

## GENOME ANNOUNCEMENT

The genus *Nocardiopsis* harbors the most abundant halophilic and halotolerant species compared to other genera in class *Actinobacteria* (1). Members of the genus *Nocardiopsis* are present in frozen soils, desert sand, compost, saline, or hypersaline habitats (marine systems, salterns, soils), and alkaline places (slag dumps, lake soils, sediments) (2). *Nocardiopsis* species produce enzymes that are cold-adapted (α-amylases), thermotolerant (α-amylases and xylanases), thermoalkalotolerant (cellulases, β-1,3-glucanases), alkalitolerant thermostable (inulinases), acid-stable (keratinase), and alkalophilic (serine proteases). Enzymes derived from *Nocardiopsis* species act on insoluble polymers such as glucans (pachyman and curdlan), keratin (feathers and prion proteins), and polyhydroxyalkanoates (2).

*N. sinuspersici* UTMC102 was discovered in sandy soil from the banks of the Arvand River, Khoramshahr, Iran (3). *N. sinuspersici* UTMC102 has the ability to produce a highly active serine alkaline protease which effectively hydrolyzes milk protein. The strain also has the genomic potential to produce a spectrum of secondary metabolites.

For genome sequencing, two libraries were constructed: a 7 to 9 kb mate-pair library, and a 450 to 550 bp paired-end library. Both libraries were sequenced using a MiSeq system. Overall, 2 × 2,867,592 MP reads (300 bp long) and 2 × 778,640 PE reads (also 300 bp long) were obtained. Nextera MP reads were processed with NxTrim (4) to separate them into proper MP/PE/single-end reads. All reads were then subjected to Trimmomatic (5) trimming. Processed reads (total coverage 106×) were assembled using SPAdes v3.8.1, discarding fragments either shorter than 1 kbp or with coverage under 50% of scaffold NOSIN_1 coverage. Scaffold NOSIN_1 is the chromosome (6,071,583 bp, 71.7% G+C content). The other 3 short contigs represent unplaced/repetitive fragments possibly belonging to the chromosome. Contig NOSIN_2 (39,728 bp, 63.1% G+C) contains 2 DEAD/DEAH helicase genes, a single *traC* conjugal transfer protein gene, 4 biosynthetic genes (including *queC*, *queE*, *queD*), and 2 transposases. Contig NOSIN_3 (4,414 bp, 67.4% G+C) contains 2 pseudogenes; database search identifies NOSIN_3 as a fragment of an NRPS gene. Contig NOSIN_4 (3,771 bp, 73.6% G+C) contains a single pseudo-gene, which yields multiple BLAST hits to NRPS genes of *Nocardiopsis dassonvillei* subsp. *dassonvillei* DSM 43111. NOSIN_4 may fill the largest (4,328 bp) of the 5 gaps in the NOSIN_1 chromosome assembly, as that gap lies within an NRPS gene. The other 4 gaps in the chromosome are inside rRNA gene clusters, between the rRNA genes.

The genome sequence was submitted to NCBI Prokaryotic Gene Annotation Pipeline. A total of 5,213 genes were identified, of them 15 rRNA genes (in 5 rRNA operons), 58 tRNA genes, and 5,056 protein coding genes, encompassing 5,052,038 nucleotides of coding regions (83% of scaffold NOSIN_1); 3,502 genes had function assignment after the annotation.

Using antiSMASH 3 (6), 63 gene clusters encoding the biosynthesis of secondary metabolites were identified, 52 of them with the ClusterFinder algorithm. In comparison, the genomes of *S. coelicolor* (7), *S. fulvissimus* (8), and *K. albida* (9) contain 20, 30, and 46 secondary metabolism gene clusters, respectively.

### Accession number(s).

This whole-genome shotgun project has been deposited at DDBJ/ENA/GenBank under the accession no. MCOK00000000. The version described in this paper is version MCOK01000000.
